# Intrauterine Administration of Autologous Platelet-Derived Growth Factor Concentrate (aka Autologous Blood Cell Derivative) Improves the Endometrial Thickness in 'Thin' Endometrium in the Frozen Embryo Transfer Cycle

**DOI:** 10.1007/s13224-022-01735-7

**Published:** 2023-04-22

**Authors:** Pratap Kumar, Anjali Mundkur, D. Sai Bhavna, Vasanthi Palanivel, Prashanth Adiga, Vidyashree G. Poojari, Shubha Rao, Rashmi Ullagaddi

**Affiliations:** 1 Department of Reproductive Medicine and Surgery, Kasturba Medical College, Manipal Academy of Higher Education, Manipal, Karnataka 576 104 India; 2 Department of Obstetrics and Gynaecology, Kasturba Medical College, Manipal Academy of Higher Education, Manipal, Karnataka 576 104 India; 3 Seragen Biotherapeutics Pvt Ltd., Bangalore Bioinnovation Centre, Helix Biotech Park Electronic City, Phase -1, Bangalore, India

**Keywords:** Autologous blood cell derivative, Platelet-rich plasma, Thin endometrium, Frozen embryo transfer, Assisted reproductive technique, In vitro fertilization

## Abstract

**Context:**

Thin endometrium during the frozen embryo transfer cycles leads to cycle cancellation. The embryo transfer cycle getting deferred is an unpleasant experience for the patients and the fertility specialist.

**Aims:**

The purpose of this study is to evaluate the effectiveness of Autologous Blood Cell Derivative (ABCD) growth factor concentrate to obtain an optimal thickness of endometrium for embryo transfer during IVF treatments, where rapid regeneration is crucial for the expected therapeutic outcome.

**Settings and Design:**

A retrospective cohort study was conducted in Manipal Assisted Reproduction Center, a referral center in Southern India.

**Methods and Material:**

Fifty-six patients with thin endometrium were administered three doses of ABCD growth factor concentrate as per the protocol after informed consent. All of them had a history of embryo transfer (ET) cancellation in frozen-thawed embryo cycles due to inadequate growth of the endometrium despite therapy with estrogens and drugs for improving uterine blood circulation.

**Results:**

The endometrium thickness during the implantation window in the patients included in the study averaged 6.48 ± 1.19 mm. After the intervention, 55 out of 56 patients (98.2%) showed a considerable change in the thickness of the endometrium layer with an average thickness of 8.48 ± 1.32 mm (< 0.0001, SE 0.233, 95% CI 1.58–2.5). Out of the 55 patients, 20 got pregnant, i.e., 36.4% pregnancy rate. Till date, thirteen pregnancies had live births (65%), three pregnancies (15%) were biochemical pregnancies, 1 (5%) was ectopic, and three pregnancies (15%) had spontaneous miscarriage before eight weeks. When we compared the endometrial thickness (EMT) in the pregnant and non-pregnant groups pre- and post-ABCD instillation, (6.47 ± 1.31 mm vs 6.48 ± 1.4 mm, *p* = 0.98 and 8.68 ± 1.32 mm vs 8.48 ± 1.32 mm, *p* value 0.59) the *p* value was not statistically significant.

**Conclusions:**

The implantation, clinical pregnancy and live birth rates were 36.4, 30 and 65%, respectively. This result is a significant improvement for patients with thin endometrium for whom we would otherwise cancel the frozen transfer. An autologous resource is a safe, readily available and inexpensive treatment modality.

## Introduction

The success of assisted reproductive technologies (ART) primarily depends on the ability of a genetically healthy embryo to be implanted in the receptive endometrium. The endometrium is an inner epithelial layer lining the uterus, a unique tissue that undergoes thousands of growth, differentiation, and detachment cycles during a woman's life [1, 2]. One of the essential criteria for the success of assisted reproductive technology (ART) programs is morphologically normal endometrium. According to available data, half to 2/3 of all implantation failures are associated with insufficient endometrial receptivity. The optimal endometrial thickness for embryo transfer is 7 mm or more [3, 4]. Insufficient endometrium thickness (< 7 mm) leads to a condition called thin endometrium resulting in low implantation, reproductive failures, ART program cancellation. A high risk of miscarriages at early stages, preterm delivery, and low birth weight children was noted in these cases [5]. The tissue of the endometrium contains various growth factors, cytokines, and lipids vital for endometrial and embryonic development, which are necessary for successful implantation. Several parameters and hormonal levels play a significant role in developing the endometrium. Angiogenesis is one such important parameter; vascular epidermal growth factor (VEGF) is an effective mediator for hormonal-dependent endometrium growth. The decreased expression of the VEGF receptor due to increased resistance in the uterine arteries results in poor endometrial growth. Hence to achieve optimal endometrial thickness, over the years, methods like hormonal manipulation are achieved with an extended dose of estrogen or improving endometrial perfusion by low dose aspirin, pentoxifylline, vitamin E, sildenafil and new modalities like Granulocyte colony-stimulating factor (G-CSF) are used. However, these methods are not proven to improve all cases with a thin endometrium. Some studies have shown better endometrial thickness (ET) after intrauterine platelet-rich plasma (PRP) [6]. The first study on PRP for treating human thin endometrium in vivo was published in 2015 [7]. Platelet-rich plasma (PRP) is a new and prospective trend in modern regenerative and reproductive medicine. This model treatment for thin endometrium is gaining attention of IVF experts. It refers to concentrating platelets in plasma to a higher level than in the whole blood [8]. The efficacy of this method is attributed to the biologically active growth factors that the platelets secrete, including pro-regenerative, proliferative, angiogenic, chemotactic, anti-inflammatory, and antiapoptotic activity. The growth factors such as VEGF, epidermal growth factor (EGF), platelet-derived growth factor (PDGF), transforming growth factor (TGF) and other cytokines that stimulate proliferation and growth are found in abundance in the PRP [9, 10].

Genetic and recombinant engineering procedures can produce growth factors, but the method seems more expensive and time-consuming. It also requires multiple doses to achieve an optimal therapeutic effect. On the contrary, ABCD or PRP is cost-effective, contains a high concentration of growth factors, is an autologous approach, thereby does not show any detrimental side effects, is non-toxic, non-allergenic and reliable. It eliminates the risk of immunological reactions and transmission infections, thus providing an excellent alternative source of treatment.

The patients included in this study were those with thin endometrium, who were treated at multiple locations with conventional treatment modalities, including hormone and vitamin therapy as well as treatment with drugs that improve blood circulation. Several IVF cycles were canceled due to insufficient endometrial thickness. They were referred to our institute, Manipal Assisted Reproduction Center, for final confirmation before recommending surrogacy or adoption. So, we decided to evaluate the effectiveness of Autologous Blood Cell Derivative (ABCD) growth factor concentrate—a next-generation PRP (Protocol developed by Seragen Biotherapeutics Pvt Ltd, Bangalore) where growth factors alone are extracted, concentrated, and suspended in cell-free serum using CDSCO approved kits.

The purpose of this study is to evaluate the effectiveness of (ABCD) growth factor concentrate to obtain an optimal thickness of endometrium for embryo transfer during IVF treatments, where rapid regeneration is crucial for the expected therapeutic outcome.

## Subjects and Method

In this retrospective cohort study conducted at our referral center in Sothern India, seventy-three patients with frozen embryo cycle cancellation due to poor endometrium thickness were enrolled in the study from June 2020–2021. Previous hormone and vitamin therapy and treatment with drugs that improve blood circulation given elsewhere did not significantly improve the thickness of the endometrium. Patients in whom the endometrium thickness during the ''implantation window'' in previous cycles (associated with hormone therapy) was ≤ 7 mm and those with poor endometrial morphology were included. The blood flow and grading of the endometrium were not done.

Out of 73, thirteen women responded to Estradiol valerate 4 mg, Vit E, Sildenafil and endometrial thickness (ET) was normalized. Three out of the remaining 60 tested positive for Covid and embryo of 1 patient degenerated. So, 56 patients were prepared for intrauterine ABCD administration. One patient out of 56 had persistent thin endometrium; hence we canceled the embryo transfer for her. Embryo transfer was successful for the remaining 55 patients.

### ABCD Administration

ABCD was administered on day 5, 6, or 7 of the menstrual cycle, or whenever the bleeding stoped completely after obtaining informed consent. Three doses of ABCD growth factor concentrate were prepared from 30 ml of peripheral blood. Dose 1 was instilled with an Intra Uterine Insemenisation catheter, and doses 2 and 3 were frozen for future use. The second dose was instilled five days after the first dose, and dose 3 was administered two days before embryo transfer. ABCD growth factor concentrate was prepared from concentrated platelets, prepared from fresh peripheral blood collected from a peripheral vein, stored in anticoagulant, and processed to separate various blood components. Whole blood was then subjected to proprietary centrifugation-based selective enrichment protocol, and the upper fraction was separated, without disturbing the RBC layer, and transferred into a sterile tube. A 100 μl sample was separated for determining platelet concentration and purity. The upper fraction was collected and centrifuged for 12 min. Platelet-poor plasma (PPP) was transferred into a sterile tube. The platelet pellet that was obtained was re-suspended in PPP. The platelet fraction alone was concentrated further from the above separations and platelets were stimulated to release cytokines and growth factors (GFs). Enriched Growth factor concentrate (ABCD) was recovered by centrifugation at 3,000 × g upto 20 min at 18 °C, then aliquoted into three 1 ml doses with PPP. The final concentration of the platelets used to prepare growth factor concentrate was approximately 600 × 10^6^.

## Statistical Analysis

The data were entered in Microsoft excel sheet. Categorical variables were expressed as numbers and percentages while continuous variables were expressed as Mean ± Standard Deviation (SD). SPSS version 21.0 (released in 2012) was used for statistical analysis. Paired t test was used for assessing the means pre- and post-intervention while unpaired t test was used to compare means of two independent groups (pregnant and non-pregnant).

## Results

After informed consent, fifty-six patients were administered three doses of ABCD™ as per the protocol.

The age of patients included in the study ranged from 20 to 42 years and averaged 34.5 ± 4.56 years, and the average BMI was 22.67 ± 3.2 kg/m^2^. Primary infertility was found in 74.5% (*n* = 41) of patients, and secondary infertility was found in 25.5% (*n* = 14). The study population of patients was homogeneous in terms of levels of LH, follicle-stimulating hormone (FSH), estradiol in the blood, and the frequency of general somatic morbidity. The endometrium thickness during the implantation window in the patients included in the study averaged 6.48 ± 1.19 mm during the ultrasound examination. Demographic, clinical, and cycle characteristics data of the patients included in the study are presented in Table 1, 2, and 3, respectively.

**Table 1:** Demographic characteristics of the cohort (*n* = 55)

Demographic parameter	Groups	*n* (%)	Mean ± SD
Age (yrs)	20–30	8(14.5)	34.5 ± 4.56
	30–40	42(76.4)	
	40–50	5 (9.1)	
BMI (kg/m^2^)	< 18.5	8 (14.5)	22.67 ± 3.2
	18.5–24.9	33 (60)	
	25–29.9	13 (23.6)	
	≥ 30	1 (1.8)

**Table 2:** Basal characteristics of the cohort (*n* = 55)

Characteristic	Groups	*n*	%
Infertility	Primary	41	74.5
	Secondary	14	25.5
Reason for IVF (one or more factors present in an individual)	Male factor	11	20
	Endometriosis	16	29.1
	PCOS	9	16.4
	Decreased ovarian reserve	30	54.5

**Table 3:** Cycle characteristics of the cohort (*n* = 55)

Characteristic	Groups	*n*	%
Type of IVF	Self	33	60
	Donor	22	40
No of prior IVF cycles	None	30	20
	1	21	29.1
	2	4	16.4
No of embryos transferred	1	17	30.9
	2	33	60
	3	5	9.1

The primary endpoint was endometrial thickness after intrauterine instillation of ABCD, and the secondary endpoint was the onset of clinical pregnancy which are presented in Table 4 and 5, respectively.

**Table 4:** Endometrial thickness (EMT)–Primary outcome (*n* = 55)

EMT (mm)	Mean ± SD	*p* value*
Pre-PRP (mm)	6.48 ± 1.19	< 0.0001 SE: 0.233 95% CI 1.58–2.5
Post-PRP (mm)	8.53 ± 1.25	

^*^Paired *t* test

**Table 5:** Secondary outcomes of the study (*n* = 55)

Secondary outcomes		*n*	%
Beta HCG negative		35	63.6
Beta HCG positive		20	36.4
	Biochemical pregnancy	3	15
	Ectopic pregnancy	1	5
	Abortion	3	15
	Live births	13	65

**Fig. 1:**
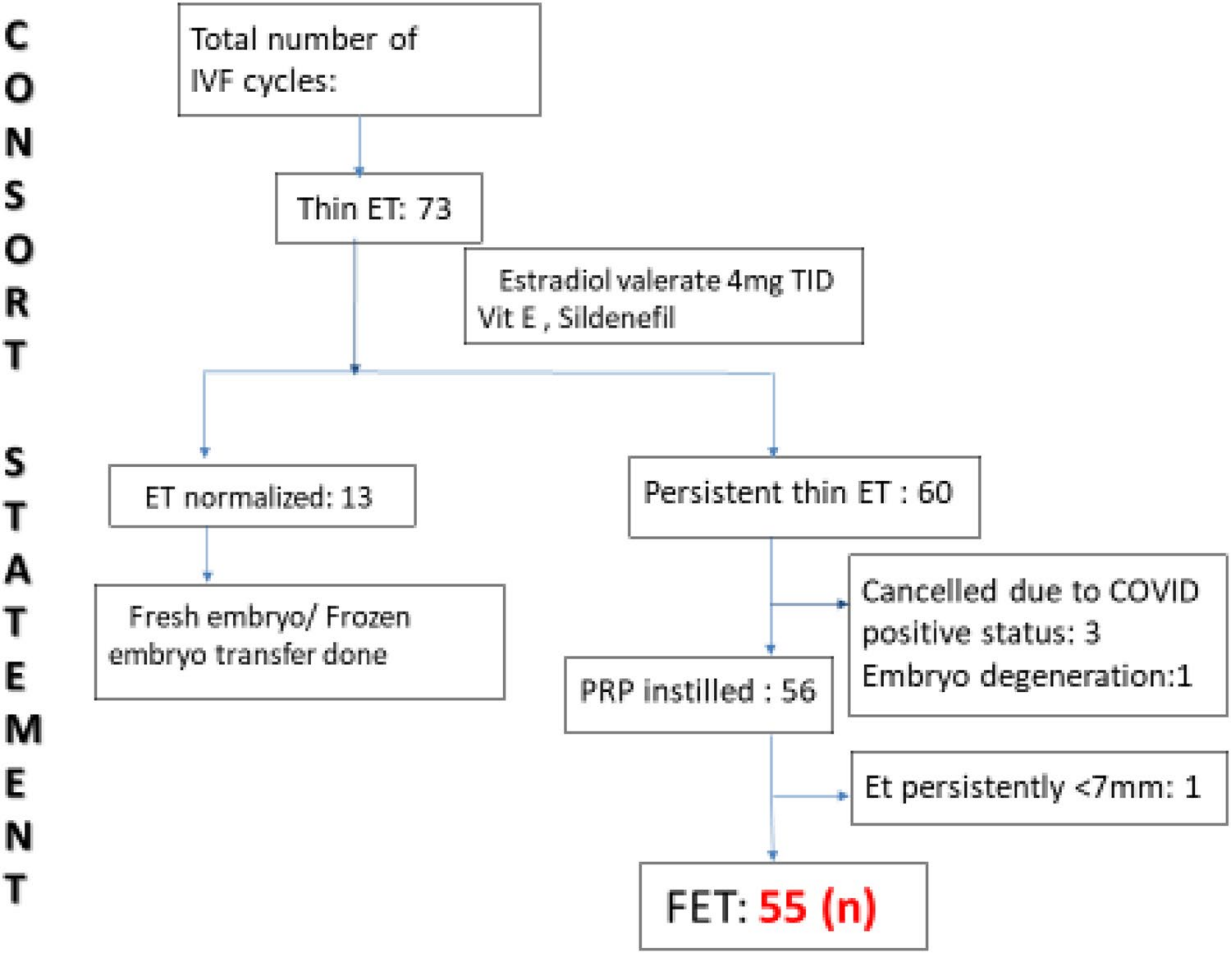
Consort statement

After the intervention, 55 out of the 56 patients included in the study showed a considerable change in the thickness of the endometrium layer and achieved an average thickness of 8.48 ± 1.32 mm from a thickness of 6.48 ± 1.19 mm (Table 4 and Fig. 1).

After embryo transfer, pregnancy occurred in 20 (36.4%) patients, and beta HCG was negative in 35 (63.6%) patients. Till date, thirteen pregnancies had live births (65%), three pregnancies (15%) were biochemical pregnancies, 1 (5%) was ectopic, and three pregnancies (15%) had spontaneous miscarriage before eight weeks (Table 5).

When comparing the increase in endometrial thickness before and after treatment in patients, we did not get a statistically significant difference between the pregnant and non-pregnant groups, (p values of 0.98 and 0.59, respectively) as presented in Table 6.

**Table 6:** Comparison of EMT in pregnant and non-pregnant patients (*n* = 55)

EMT categories	Pregnant (*n* = 20)	Non-pregnant (*n* = 35)	*p* value*
Pre-ABCD EMT (mm)	6.47 ± 1.31	6.48 ± 1.4	0.98
Post-ABCD EMT (mm)	8.68 ± 1.32	8.48 ± 1.32	0.59

^*^Independent samples *t* test

The follow-up of the patients was as follows:

There were 24 Day 3 transfers and 31 Day 5 transfers. Out of 20 patients with pregnancy, 9 were Day 3 transfer and 11 were Day 5 transfer. Grade of the embryo was not analyzed. Post embryo transfer, patients received vaginal 8% progesterone gel twice a day till positive pregnancy test and continued till 8 weeks. Estradiol valerate 2 mg was given thrice a day till positive pregnancy test. Patient’s positive for Antiphospholipid antibody received low molecular weight heparin.

## Discussion

The main objective of this study was to determine the regenerative effects of ABCD growth factor concentrate on thin endometrium and implantation rate. Over the years, methods like hormonal manipulation (giving an extended dose of estrogen) or improving endometrial perfusion by low dose aspirin, pentoxifylline, vitamin E, sildenafil and new modalities like Granulocyte colony-stimulating factor (G-CSF) are being used for endometrial expansion. However, these methods cannot improve all cases with thin endometrium. The first study on PRP for treating human thin endometrium in vivo was published in 2015 [7]. Some studies have shown better endometrial thickness (EMT) after intrauterine platelet-rich plasma (PRP) [6–8, 11–13].

Our study aimed at analyzing the EMT improvement of patients with thin endometrium with a history of multiple FET cycle cancellations due to thin endometrium (< 7 mm), those who underwent other methods of EMT enhancement including Vitamin E & Sildenafil and found no considerable improvement which led to repeated cancellation of embryo transfer cycles. These patients were left with the option of surrogacy as their only resolve.

In these 56 patients treated with ABCD growth factor concentrate, the improvement in the endometrial thickness was seen in 55 patients, i.e., 98.2% rate of improvement in EMT. 36.4% of the patients were beta HCG-positive, out of which 65% delivered live babies. Successful embryo implantation is a complex process that depends on a coordinated cross-talk between the endometrial factors and the embryo itself. Good embryo quality is one of the key factors and numerous variables, like lifestyle, hormonal changes, and several other environmental and molecular factors affect it [14]. The implantation rates expressed in positive pregnancy tests and live birth in both complex groups of patients presented satisfactory results, matching the findings obtained in the few previous serial clinical studies on the matter.

Although PRP is widely applied in different clinical areas, preparing PRP is not yet standardized [15]. Methods described for PRP preparation for intrauterine administration are not precise, and they seem to be similar to those used to prepare peripheral blood-derived products. Thus, it is possible that when preparing PRP from whole blood, the final plasma product used as PRP contains platelets in addition to the essential cytokines and growth factors released by the peripheral blood mononuclear cells present in the whole blood which are majorly pro-inflammatory. Therefore, the platelet quantification and growth factor contents are not defined. Precise knowledge of the identity, concentration, effects of the individual blood factors, their origin, whether platelets with or without blood mononuclear cells will significantly improve and make results obtained in fertility treatments more repeatable. We attempted to create the fertility and implantation-friendly version of platelet derivative for rapid action as events during IVF are time-bound. The optimal biological effect occurs when a lymphocyte-free platelet concentration of approximately 600 × 10^6^ per ml is used. The cell-free growth factor concentrate made it possible to achieve optimal endometrial thickness in almost all the patients for embryo transfer; the difference in endometrial thickness before and after therapy was statistically significant; the difference in the frequency of cancellation of embryo transfer cycles was also statistically significant with satisfactory pregnancy and live birth rate. Based on our results, it can be assumed that the use of ABCD improves the outcomes of ART programs in patients with "thin" endometrium since this cohort of patients is very complex and there are no effective methods for preparing the "thin" endometrium for embryo transfer. Importantly, ABCD is made from autologous blood, which excludes allergic reactions and the transmission of infectious diseases.

ABCD growth factor concentrate is a biological product derived from pure platelet concentrates devoid of other cellular components, customized exclusively to be very effective in Infertility where rapid regeneration is crucial for the expected therapeutic outcome. The main advantage of ABCD growth factor concentrate, which is derived from patient’s own platelet concentrates stimulated to secrete growth factors many folds higher than physiological level, is capable of triggering the tissue regeneration and remodeling in a fast-track mode which is the critical requirement in a specialty like infertility where every event is time-bound and the faster regeneration (< 15 days) is the key to successful cycle completion. ABCD growth factor concentrate attributes to other advantages like multiple doses from single blood collection and consistency of doses, thereby providing convenience to the fertility care provider and patient contributing to seamless incorporation into IVF protocols. This promotes us to conduct bigger prospective studies including endometrial vascularity and type prior to instillation of the growth factor concentrate to improve endometrial thickness.

## Conclusion

Even after four decades of reproductive technologies, the endometrium with thickness less than 7 mm and poor receptivity is a condition that has created confusion among ART clinicians worldwide and remains an unsolved problem. Despite enormous conventional treatment modalities accessible today, it is still not easy to furnish a realistic, corroborative approach that guides the clinician on improving refractory endometrium. The present study was conducted as a retrospective cohort study to analyze the effects of ABCD treatment on refractory thin endometrium. The result of this intervention is a significant improvement for patients with thin endometrium for whom we would otherwise cancel the frozen transfer. The clinical pregnancy and live birth rates reached 85 and 65%, respectively. This study of improving endometrial thickness by ABCD growth factor concentrate, where a biological product derived from pure platelet concentrates devoid of other pro-inflammatory cellular components, yielded promising results, has created new options for the use of ABCD growth factor concentrate in infertile women with previously canceled cycles due to very poor refractory thin endometrium. Being an autologous resource, it is a safe, readily available, and inexpensive treatment modality. Hence, it is recommended to be incorporated in the different endometrial preparation protocols during IVF procedures, even in those where an HRT-free natural cycle is preferred.

Further studies are recommended in terms of the number of populations under study and the time frame evaluated, the molecular basis of this ready to act growth factor concentrate treatment, as well as the approach of comparative studies between drugs and autologous preparations, to reveal the exact mechanism and to obtain more solid evidence for creating effective therapeutic alternatives for refractory endometrium.

## Abbreviations

ABCD: Autologous blood cell derivative
TM: Trade mark
PRP: Platelet-rich plasma
FET: Frozen embryo transfer

## References

[1] Gargett CE (2007). Uterine stem cells: what is the evidence?. Human Reprod Update..

[2] Maruyama T, Masuda H, Ono M (2010). Human uterine stem/progenitor cells: their possible role in uterine physiology and pathology. Reproduction.

[3] El-Toukhy T, Coomarasamy A, Khairy M (2008). The relationship between endometrial thickness and outcome of medicated frozen embryo replacement cycles. Fertil Steril.

[4] Richter KS, Bugge KR, Bromer JG, Levy MJ (2007). Relationship between endometrial thickness and embryo implantation, based on 1294 cycles of in vitro fertilization with transfer of two blastocyst-stage embryos. Fertil Steril.

[5] Mouhayar Y, Franasiak JM, Sharara FI (2019). Obstetrical complications of thin endometrium in assisted reproductive technologies: a systematic review. J Assist Reprod Genet.

[6] Tandulwadkar SR, Naralkar MV, Surana AD (2017). Autologous intrauterine platelet-rich plasma instillation for suboptimal endometrium in frozen embryo transfer cycles: a pilot study. J Human Reprod Sci.

[7] Chang Y, Li J, Chen Y (2015). Autologous platelet-rich plasma promotes endometrial growth and improves pregnancy outcome during in vitro fertilization. Int J Clin Exp Med.

[8] Malanga GA, Goldin M (2014). PRP: review of the current evidence for musculoskeletal conditions. Curr Phys Med Rehabil Rep.

[9] Zadehmodarres S, Salehpour S, Saharkhiz N, Nazari L (2017). Treatment of thin endometrium with autologous platelet-rich plasma: a pilot study. JBRA Assist Reprod.

[10] Agarwal M, Mettler L, Jain S (2020). Management of a thin endometrium by hysteroscopic installation of platelet-rich plasma into the endomyometrial junction: a pilot study. J Clin Med.

[11] Colombo GVL, Fanton V, Sosa D (2017). Use of platelet rich plasma in human infertility. J Biol Regul Homeost Agents.

[12] Molina A, Sánchez J, Sánchez W, Vielma V (2018). Platelet-rich plasma as an adjuvant in the endometrial preparation of patients with refractory endometrium. JABRA Assist Reprod.

[13] Nazari L, Salehpour S, Hoseini S (2019). Effects of autologous platelet-rich plasma on endometrial expansion in patients undergoing frozen-thawed embryo transfer: a double-blind RCT. Int J Reprod BioMed.

[14] Tiitinen A (2019). Single embryo transfer: why and how to identify the embryo with the best developmental potential. Best Pract Res Clin Endocrinol Metab.

[15] Amable PR, Carias RB, Teixeira MV (2013). Platelet-rich plasma preparation for regenerative medicine: optimization and quantification of cytokines and growth factors. Stem Cell Res Ther.

